# Medicine of the Future

**Published:** 1887-01

**Authors:** 


Medicine of the Future. By Austin Flint, M. D., LL.D. New York: D. Appleton and Company. 1886.

This is the address which was prepared by the author and was to have been read by him at the last meeting of the British Medical Association. Standing on the pinnacle of fame, at the end of a long and excellent career, he peers into the future, and then, like the great law-giver who viewed the land he could not tread, lies down and closes his eyes forever. He sees progress in every department and predicts for medicine a glorious future. The address is neatly gotten up and contains as a frontispiece a faithful picture of the author.

